# Impact of prolonged treatment with high-dose ciprofloxacin on human gut flora: a case report

**DOI:** 10.1186/1752-1947-4-111

**Published:** 2010-04-21

**Authors:** Irene S Kourbeti, Dimitrios E Alegakis, Sofia Maraki, George Samonis

**Affiliations:** 1 Department of Internal Medicine, University Hospital of Heraklion, Voutes, Crete, 71110, Greece; 2 Department of Clinical Bacteriology, Parasitology, Zoonoses and Geographical Medicine, University Hospital of Heraklion, Voutes, Crete, 71110, Greece

## Abstract

**Introduction:**

Ciprofloxacin is a commonly marketed fluoroquinolone. It is not effective against obligate anaerobes, hence it is considered unlikely to have an impact on colonic microflora. We report the case of a patient who received prolonged treatment with high-dose ciprofloxacin for extensive pelvic osteomyelitis. We also report on the medication's effects on his bowel flora. To the best of our knowledge, this is the first report discussing the effects of prolonged administration of a quinolone on microbial flora.

**Case presentation:**

A 62-year-old Caucasian man with diabetes presented with low back pain of four months' duration. A magnetic resonance imaging of his pelvis revealed sacroiliitis and extensive pelvic osteomyelitis. *Pseudomonas aeruginosa*, which is susceptible to ciprofloxacin, was noted as the offending pathogen. After seven weeks of intravenous treatment, he was prescribed with high-dose oral ciprofloxacin that he continued to take for the next 20 months. Quantitative stool cultures of our patient were obtained a month later as well as at the end of his treatment to record his corresponding sensitivities to the medication. The Gram-negative population of his bowel flora was restored fully upon the discontinuation of this medication. The Gram-negative population was shown to be fully sensitive to ciprofloxacin. His yeast levels were also found to be slightly increased, and no growth of resistant enterococci was noted.

**Conclusion:**

The findings of this case report suggest that long term and high dose ciprofloxacin administration might be safe in preventing the risk of colonization with resistant Gram negative pathogens, overgrowth of anaerobes and the development of resistant enterococci.

## Introduction

Ciprofloxacin remains the most potent of the marketed fluoroquinolones. Its spectrum includes aerobic Gram-negative bacilli, particularly Enterobacteriaceae. It is effective, even under unfavorably anaerobic conditions involving *Haemophilus spp., Neisseria spp. *and non-enteric Gram-negative bacilli, such as *Pseudomonas aeruginosa *[1].

However, ciprofloxacin is not considered to be clinically effective against obligate anaerobes. Since the colonic microflora consists of a vast majority of anaerobes, conventional wisdom holds that ciprofloxacin is unlikely to have a major impact on colonic microflora [2]. We report the case of a patient who received prolonged treatment with high-dose ciprofloxacin for extensive pelvic osteomyelitis.

## Case presentation

A 62-year-old Caucasian man with diabetes presented with low back pain that he had been experiencing for four months.

An MRI of his pelvis, including his right sacroiliac joint and pelvic floor muscles, revealed osteomyelitis of the pubic symphysis and extensive pelvic osteomyelitis.

Pus obtained from his right sacroiliac joint, as well as several pieces of his sacrum, grew *P. aeruginosa*.

As is usually done in severe infection with Pseudomonas, our patient was given double antibiotic therapy for almost two months. His treatment consisted of two types of antibiotics to which the pathogen was susceptible (seven weeks of intravenous ciprofloxacin and piperacillin and/or tazobactam).

At present, available data are limited to extensive, life-threatening infections such as in the case of our patient. The literature, however, consists of cases supporting the long-term administration of ciprofloxacin monotherapy in bone infections caused by *Pseudomonas *after an initial period of combination therapy [3]. Because of the high probability of infection recurrence, our patient continued with high-dose oral ciprofloxacin (1 gram twice a day) for 20 months. Repeat MRIs confirmed a marked improvement of his inflammation, which subsequently led us to stop his antibiotic therapy.

The quantitative stool cultures of our patient were studied after 20 months of treatment with oral ciprofloxacin. After incubation at 37°C under aerobic and anaerobic conditions for two to five days, qualitative and quantitative analyses of the cultures were performed. The cultures yielded 3 × 10^6 ^CFU/mL *of Escherichia coli*, 10^7 ^CFU/mL *of Enterococcus faecium*, 2 × 10^4 ^CFU/mL *Staphylococcus epidermidis*, 6 × 10^4 ^CFU/mL *of Corynebacterium *group A, 2 × 10^3 ^CFU/mL of *Bacillus stearothermophilus*, 10^10 ^CFU/mL of *Bacteroides uniformis*, 2 × 10^9 ^CFU/mL of *Prevotella oralis*, 5 × 10^8 ^CFU/mL of *Prevotella loescheii *and 3 × 10^4 ^CFU/mL of *Candida albicans*.

Susceptibilities of aerobes and anaerobes to antibiotics were performed for five randomly selected colonies of all bacterial isolates using the disk diffusion method recommended by the Clinical and Laboratory Standards Institute (CLSI) [4]. As expected, *E. coli *was resistant to ciprofloxacin and the rest of the fluoroquinolones tested. The population of *E. faecium*, meanwhile, was resistant to the fluoroquinolones but sensitive to vancomycin. Repeat stool cultures were taken one month after the discontinuation of our patient's antibiotics treatment, and the same method was performed. There was a marked increase in the Enterobacteriaceae population with *E. coli *at up to 5 × 10^8 ^CFU/mL. The Gram-negative flora was enriched with *Klebsiella pneumoniae *(5 × 10^6 ^CFU/mL) and *Enterobacter cloacae *(6 × 10^7 ^CFU/mL). The susceptibility of *E. coli *to fluoroquinolones was restored, while *K. pneumoniae *and *E. cloacae *were also found to be fully susceptible to fluoroquinolones. A small population of enterococci (*E. faecalis *1.5 × 10^6 ^CFU/mL and *E. avium *2 × 10^6 ^CFU/mL) was reported as being intermediately sensitive to the fluoroquinolones but fully sensitive to vancomycin. The yeast population decreased from 3 × 10^4 ^CFU/mL to 8 × 10^3 ^CFU/mL, while the anaerobic population remained basically unchanged.

## Discussion

The huge number of anaerobes in the human gut microflora prevents its colonisation and the invasion of tissues by exogenous pathogenic bacteria including the opportunists. A disruption of this balance by antibiotics produces profound effects on the protective barrier and might result in the overgrowth of pathogens and also in the development of life-threatening infections. Controlling the growth of opportunistic microorganisms is termed "colonization resistance" [5-7].

The common finding in our patient, the *in vitro* experiments and the clinical trials, is the significant reduction in the levels of *E. coli*[2,5,8]. The *E. coli *population was resistant to quinolones but no extended spectrum resistance to β-lactams, especially cephalosporins, was detected.

Quinolones are notorious for suppressing or even eliminating intestinal Enterobacteriaceae [9]. The effect of antibiotics on the enterococci and the anaerobic population varies in different clinical trials, but most of them have not been used in real situations with prolonged administrations of high drug doses.

It seems that aside from norfloxacin, quinolones have produced a minor effect on them [2,8,9]. The overgrowth of resistant bacteria has not generally been seen [9], nor has susceptibility to ciprofloxacin for *E. coli *and enterococci been universal [2]. Studies in patients with cirrhosis who received quinolones for spontaneous bacterial peritonitis prophylaxis have demonstrated contradictory results. These studies, however, involved patients who took quinolones daily for a short period of time [10,11].

## Conclusion

Despite the significant reduction and resistance in the *E. coli *population and the suppression of the rest of the Enterobacteriaceae at the end of the ciprofloxacin treatment of our patient, we found neither substantial overgrowth of resistant bacteria or yeasts nor significant changes in the enterococci or anaerobic population. In that sense, our findings concur with the ones by Edlund and Nord [9]. It should be noted that the sensitivity of *E. coli *to ciprofloxacin was restored one month after the drug therapy of our patient was discontinued. This was confirmed by the findings we made regarding the cultures performed in the same laboratory and by the same methodology one month after he was started on the treatment and again at the end of it.

To the best of our knowledge, this is the first reported case describing the effects of long-term, high-dose ciprofloxacin administration on colonic flora. The important conclusions drawn from this case report are the fast recovery of the intestinal Gram-negative flora and its complete sensitivity to ciprofloxacin after the discontinuation of the antibiotic, the sustained sensitivity of enterococci to vancomycin, and the preserved anaerobic population despite a long-term administration of antibiotics. The impact of anti-pseudomonas penicillin on the colonic flora cannot be ignored. However, there was a lapse of 20 months between its discontinuation and the assessment of the sensitivities of the colonic flora. Therefore, we consider that the piperacillin and/or tazobactam impact during the time of testing was negligible. Unfortunately, no sensitivity tests were done immediately after the antipseudomonal penicillin was discontinued.

The findings of this case report suggest that long term and high dose ciprofloxacin administration might be safe in preventing the risk of colonization with resistant Gram negative pathogens, overgrowth of anaerobes and the development of resistant enterococci.

## Consent

Written informed consent was obtained from the patient for publication of this case report and any accompanying images. A copy of the written consent is available for review by the Editor-in-Chief of this journal.

## Competing interests

The authors declare that they have no competing interests.

## Authors' contributions

ISK and DEA took care of our patient, reviewed the literature, and wrote most of the manuscript. SM performed the microbiological studies. GS was the senior consultant in our patient's care. He also critically reviewed the manuscript. All authors read and approved the final manuscript.
